# The Click Reaction as an Efficient Tool for the Construction of Macrocyclic Structures

**DOI:** 10.3390/molecules18089512

**Published:** 2013-08-08

**Authors:** Dario Pasini

**Affiliations:** 1 Department of Chemistry, University of Pavia, Viale Taramelli, 10-27100 Pavia, Italy; E-Mail: dario.pasini@unipv.it; Tel.: +39-0382-987835; Fax: +39-0382-987323; 2 INSTM Research Unit, University of Pavia, Viale Taramelli, 10-27100 Pavia, Italy

**Keywords:** CuAAC, click chemistry, triazoles, macrocycles, chemical sensors, molecular recognition

## Abstract

The Cu(I)-catalyzed azide-alkyne cycloaddition (CuAAC, known as the click reaction) is an established tool used for the construction of complex molecular architectures. Given its efficiency it has been widely applied for bioconjugation, polymer and dendrimer synthesis. More recently, this reaction has been utilized for the efficient formation of rigid or shape-persistent, preorganized macrocyclic species. This strategy also allows the installment of useful functionalities, in the form of polar and function-rich 1,2,3-triazole moieties, directly embedded in the macrocyclic structures. This review analyzes the state of the art in this context, and provides some elements of perspective for future applications.

## 1. Introduction

Macrocycles are defined as “cyclic macromolecules or macromolecular cyclic portions of a molecule” [1]. The chemistry of macrocycles goes back a long way, but it is safe to say that the field has greatly expanded in breadth and scope since the advent of the “chemistry beyond the molecule” [2,3]; in fact, the birth of supramolecular chemistry is traditionally associated to the publication of the synthesis of crown ethers by Charles Pedersen almost 50 years ago [4]. Since then, the use of macrocycles as hosts capable of recognition of specific guests, and in conjunction with suitable assembly strategies for the construction of a large variety of nanoscale structures, has experienced increasing interest [5,6].

The synthesis of macrocycles has evolved in recent years by developing concepts pertaining to different traditional areas of chemistry. We wish to summarize here three key aspects as follows:

(a) the use of high dilution conditions has greatly helped to improve yields in the cyclization process; this tool statistically favors ring closing *vs.* polymerization reaction, either in single chains containing complementarily reactive chain end functionalities (AB; A reacting with B), or in chains containing complementary reactive functionalities (AA with BB) [5,6,7];
(b) template-directed syntheses: it is the exploitation of coherently designed guest systems, recognized by the forming host, which can effectively act as templates during the reaction, in order to pre-organize the host-guest system for the macrocyclization reaction (forming one or more new covalent bonds), facilitating the desired chemistry [5,6].
(c) Dynamic combinatorial chemistry, as a method for the generation of new molecules by means of reversible reactions between simple building blocks under thermodynamic control. In a dynamic combinatorial library (DCL) all constituents are in equilibrium, and their distribution is determined by their thermodynamic stability within the DCL. This tool has been used with success for the construction, amongst others, of macrocyclic structures [8].

The key issue at stake, whatever strategy is used, is to utilize efficient, high-yielding, functional-group tolerant, orthogonal [9] organic chemistry for the ring-closing step (the macrocylization reaction). Cu(I)-catalyzed azide-alkyne cycloaddition (CuAAC) reactions often have all the required characteristics. In fact, 1,3-dipolar cycloadditions between azides and terminal alkynes were studied in detail by Huisgen and coworkers almost 50 years ago [10]. The reaction is thermodynamically favored, but it requires heating, and it does not show regioselectivity (both 1,4-disubstitued and 1,5-disubstituted-1,2,3-triazoles are formed) [11]. The catalyzed version of this synthetic methodology was introduced in the early 2000s, when seminal papers by Sharpless and Meldal [12,13] showed that catalysis by Cu(I) species greatly enhanced the reactivity (up to 10^7^ rate enhancements), and the reaction can be carried out at room temperature; furthermore, the catalyzed reaction is extremely regioselective towards the formation of the 1,4-disubstituted triazole (over the 1,5-disubstituted isomer).

**Scheme 1:**
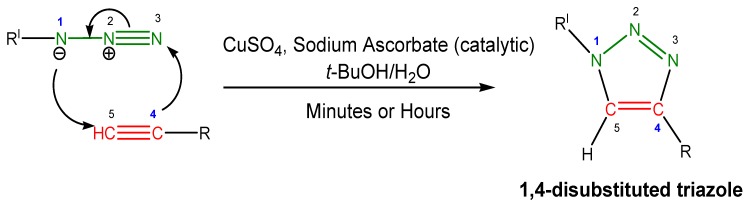
Prototypical conditions for the Cu(I)-catalyzed azide alkyne cycloaddition (CuAAC) reaction, a 1,3-dipolar cycloaddition, and the resulting product.

Sharpless initially utilized the generation of catalytic Cu(I) by the *in situ* addition of the reducing sodium ascorbate to Cu(II) salts, in a mixture of solvents capable of dissolving both the organic reactants and the inorganic counterparts (Scheme 1). The reaction, by all means, falls into the category of “click chemistry”. In fact, Sharpless [14] defined the term “click chemistry” as follows: “The reaction must be modular, wide in scope, give very high yields, generate only inoffensive byproducts that can be removed by nonchromatographic methods[…] The required process characteristics include simple reaction conditions (ideally, the process should be insensitive to oxygen and water), readily available starting materials and reagents, the use of no solvent or a solvent that is benign (such as water) or easily removed, and simple product isolation.[…] Click processes proceed rapidly to completion and also tend to be highly selective for a single product: we think of these reactions as being springloaded for a single trajectory.

By virtue of its simplicity, and its water-tolerant operational conditions, click chemistry has quickly become an established tool for the construction of complex molecular architectures. In particular, the functionalization of macromolecular structures, the synthesis of dendrimers, and the conjugation between natural and artificial macromolecules has been targeted using CuAAC [15,16,17,18,19,20,21,22]. A copper-free variant of CuAAC has also been successfully developed and it is gaining increasing popularity [23].

In this review, we will highlight recent examples of the use of the CuAAC reaction as the *macrocyclization reaction* in several contexts. We exclude from the present review: (a) cyclic structures in which part of the cyclic connection is formed by noncovalent bonds [24]; (b) the postmodification with CuAAC click chemistry of preformed macrocycles in order to introduce triazoles for functional applications [25,26]; (c) coordination polymers, or metal-organic frameworks, formed by “clicked” struts or ligands [27]. The review is structured in several sections according to the different classes of macrocycles obtained.

## 2. Peptide- and Sugar-Containing Click Macrocycles

Macrocyclic peptides are attractive molecular scaffolds in order to develop bioactive compounds with the capability to modulate biomolecular interactions. In these cyclic frameworks, the high degree of functional complexity which is usually achievable within simpler oligopeptides is combined with restricted conformational flexibility, making them suited to achieve selective and tight binding to extended biomolecular interfaces, such as those mediating protein–protein and protein–nucleic acid complex formation [28]. Some of the enhanced features and properties exhibited by conformationally constrained peptide-based ligands, compared to linear peptides, include enhanced cell permeability [29] and higher affinity towards the target biomolecule.

Recent developments in the use of the CuAAC reactions in the synthesis, modification, and conformational control of peptidomimetic oligomers were reviewed in 2009 [30]. Both synthetic and biosynthetic methods have been implemented to afford peptides in cyclic or conformationally constrained configurations. Recent reports use CuAAC click chemistry in combination to a genetic encoding strategy for the synthesis of libraries of cyclic peptides.

**Scheme 2:**
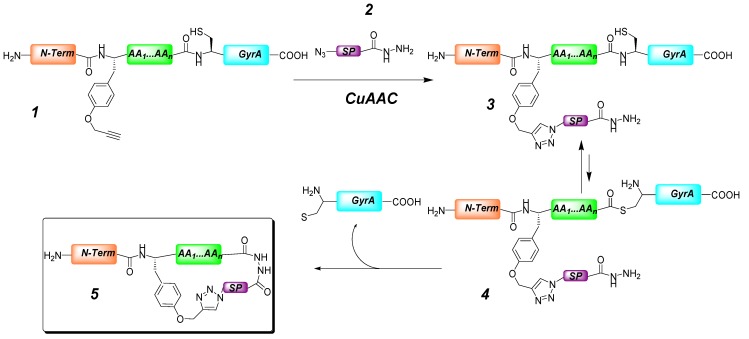
Strategy for the modular synthesis of macrocyclic organo-peptide hybrids ***5***. The starting linear polypeptides comprise an *N*-terminal tail (orange), *O*-propargyl tyrosine, a target sequence (green), and a GyrA intein segment (light blue). Macrocyclization occurs upon coupling of this protein to a synthetic precursor (purple) by concomitant CuAAC and thioester-hydrazide coupling [31].

The group of Fasan has reported a new method for constructing conformationally constrained organo-peptide hybrids by combining a genetically encoded polypeptide and a synthetic precursor (Scheme 2) [31]. In their strategy, an alkyne-bearing unnatural amino acid was incorporated within the *N*-terminal portion (orange box in Scheme 2) of the linear hybrid **1**, formed by an intein protein (a protein segment able to excise itself, light blue box) fused to a chosen polypeptide (green box). The synthetic precursors (purple box, structure **2**) were aromatic units containing suitable complementary functionalities (to alkyne and thioesters of the hydrid strands) in the form of azide and hydrazide moieties. The bioorthogonal functionality is a thioester bond formed *in situ* by action of the thiol group of the *N*-termini cysteine residue of the intein protein on its amide connectivity (see structure **4** in Scheme 2). The CuAAC reaction between the azide of the synthetic precursor and the alkyne moiety of the unnatural aminoacid, coupled with the hydrazide thioester reaction, afforded the organo-peptide macrocycle **5** with the concomitant release of the intein protein. The CuAAC protocol used proceeded quantitatively with the various biosynthetic precursors within minutes; prototypical conditions for the click chemistry (CuSO_4_, sodium ascorbate in buffered aqueous solutions) were used, with addition of EDTA (ethylenediaminetetraacetic acid) to facilitate copper removal. The authors could easily monitor the reactions using MALDI-TOF spectra acquired immediately after the coupling reaction.

One further example of the efficiency and applicability of CuAAC methodologies to peptide chemistry have been given recently by the group of Liu [32]. They have successfully implemented a chemical system in which DNA templates the generation of sequence-defined synthetic polymers (modified cyclic and acyclic oligopeptides), with no structural similarity to the templating nucleic acid moieties. The building blocks can be varied into backbone structural diversity (polyethylene glycols, α-peptides and β-peptides). The authors identified the CuAAC reaction and the AA/BB substrate architecture as key factors to achieve efficient translation. Also in this paper, prototypical conditions for the click chemistry (CuSO_4_, sodium ascorbate) were used, with overnight incubation at 4 °C and salt removal with short column chromatography. The CuAAC reaction has been reported recently for cyclization reactions of oligopeptides carried out directly on the resins, thus in heterogeneous conditions which always characterize solid phase synthesis [33,34,35]. In all these reports, the CuAAC reaction yields are high.

**Figure 1:**
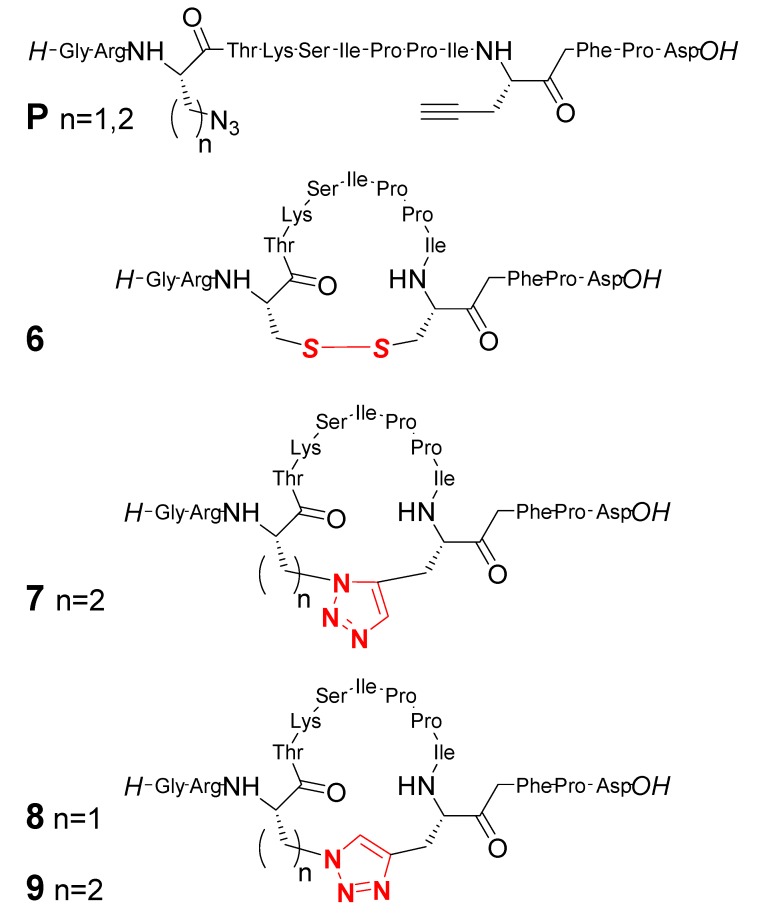
Cyclic peptides obtained by CuAAC or RuAAC click chemistries incorporating 1,4- or 1,5-disubtituted triazole linkages as trypsin inhibitors analogue of **6** [36].

The Kolmar group has demonstrated the utility of a 1,2,3-triazole bridge as a disulfide replacement [36]. The efficient *in-vitro* generation of disulfide bonds is still a challenge in contemporary biochemistry; in fact, it is usually achieved in peptides post-synthetically by means of the use of mediators such as air oxygen. It is particularly challenging in cysteine-rich oligopeptides, for which the regiospecific formation of one out of several disulfide bonds is not trivial to control. 1,2,3-Triazole can be expected to act as efficient disulfide surrogates as they are redox stable, and dissimilar to common building blocks of nature, so that improved pharmacokinetic properties can be anticipated. In their communication, the authors designed a series of triazole analogues of a monocyclic variant of the sunflower trypsin inhibitor-I (SFTI-1, cyclic peptide **6** in Figure 1). The analogues **7**–**9** have been obtained through the cyclization of linear peptides, bearing sequences of aminoacids which include unnatural azide and alkyne-functionalized ones (precursors **P** in Figure 1). The authors used both Cu(I) and the Ru(II) catalyzed versions of the azide alkyne cycloaddition reaction. This latter version, introduced by Sharpless [37] subsequent to CuAAC, allows for the regioselective formation of 1,5-disubstituted triazole derivatives from azides and terminal alkynes. Although the RuAAC variant gathers much less popularity than CuAAC, it has been used with success in the context of different scenarios, and we refer the reader to selected recent examples [38,39,40,41,42,43]. CuAAC could be carried out using solution phase synthesis techniques in solution: cyclic peptides **8** and **9** (Figure 1) were obtained using classic CuAAC conditions [Cu(SO_4_)_2_, sodium ascorbate] and high dilution and isolated in modest yields (10%–20%) after purification by HPLC. RuAAC was carried out after anchoring the linear precursors on a solid phase, eventually achieving the 1,5-disubstituted triazole-containing cyclic peptide **7**, although in low yields (2%). The authors stated that RuAAC did not work satisfactorily using solution phase synthesis techniques. The low yields are likely the consequence of the high degree of complexity and of conformational flexibility of the precursors to be cyclized, rather than of the inefficient protocols/conditions utilized for the CuAAC reaction.

The design considerations were substantiated by energy-minimized 3D models of all variants of **6**. From these calculations, the 1,5-disubstituted triazole bridge of **7** resulted as more capable, when compared to the 1,4-sustitution pattern on the triazole present in substrates **8** and **9**, of optimal structural similarity and almost perfect superposition with cyclic peptide skeleton of **6**. Indeed, inhibitory activity studies showed that, whereas analogue **7** essentially retained the activity of **6**, both **8** and **9** are at least two orders of magnitude less effective than **6**. This work demonstrates the feasibility of the substitution of a disulfide bridge by a triazole linkage, and in general the broad applicability of AAC reactions for cyclopeptide mimic.

**Figure 2:**
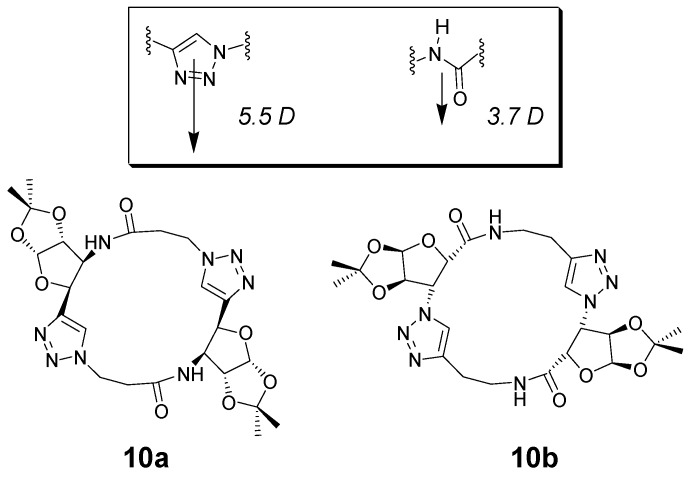
Regioisomeric triazole-containing cyclic peptides composed of *cis*-β-furanoids and β-alanines [48].

One of the most appealing areas of research related to macrocyclic chemistry is their use as building blocks for the assembly of nanotubes [44,45,46]. Cyclic d,l-α-peptides, and more recently cyclic β^3^-peptides, have been shown to assemble into tubular structures through the association of complementary and suitably positioned amide hydrogen bonding units within the cyclic backbone [47]. Triazoles can be considered as amide bond analogues; they are, in fact, suitable mimics of the amide bond in terms of polarity, as well as hydrogen bond donating and accepting ability (Figure 2).

In recent work, the group of Chattoparday has reported cyclic peptides able to assemble in oriented nanotubes [48,49]. The triazoles embedded within the cyclic structures were obtained though CuAAC reactions between alkyne and azide moieties of elaborated precursors, containing *cis*-β-furanoid sugars and β-alanine moieties, although the click reaction was not used in the cyclization step. The reactions were carried out in classical CuAAC conditions [Cu(SO_4_)_2_, sodium ascorbate] in mixtures of organic solvents and water. Two regioisomeric products (**10a** and **10b** in Figure 2) were designed and separately obtained. Changes in the packing and self-assembly of these macrocycles, as a consequence of the subtle structural changes in their regioisomeric structures, resulted in different polarity of the nanotubes due to different orientation of functional groups. These macrocycles are effective model systems for artificial ion channels, and their unidirectionality in terms of dipole moments augurs well for functional applications of this new class of peptidomimetic macrocycles. Work in this area has also been recently reported by the groups of Abell and James [50,51].

**Scheme 3:**
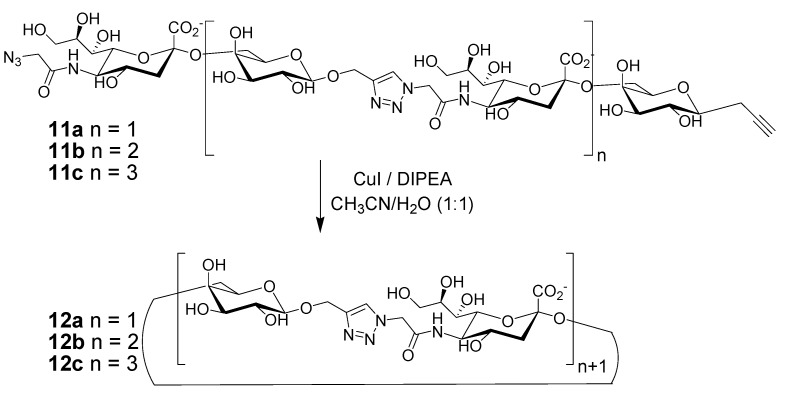
Synthesis of click macrocycles through the cyclization of oligosaccharide linear monomers of varying lengths [56].

The use of CuAAC click chemistry for the synthesis of sugar-containing macrocycles has been recently reported by several groups, and very interesting properties have been demonstrated for these derivatives [52,53,54,55], such as metal complexation ability, and the possibility to address simply stereochemical diversity. The Chen group [56] reported the synthesis and characterization of structurally well-defined macrocyclic oligosaccharides of various dimensions. Their strategy involves a series of chemical, but also highly efficient chemoenzymatic methods, for the preparation of the precursor oligosaccharides **11** (Scheme 3), incorporating an azido-containing sialic acid at the nonreducing end and a propargyl group at the reducing end. The CuAAC macrocyclization reaction in a mixture of organic solvent/H_2_O was carried out using “non classical” conditions, by means of directly providing Cu(I) as the catalyst, instead of generating it *in situ*. Yields in the range 30%–80% could be obtained: such yields have to be considered outstanding, given the relative flexibility and poor preorganization of the substrates. The ionic macrocycles **12** possess high solubility in water and, like cyclodextrins, can encapsulate hydrophobic aromatic small molecules in a size-dependent manner.

## 3. Click Macrocycles for Anion Binding and Supramolecular Recognition

**Figure 3:**
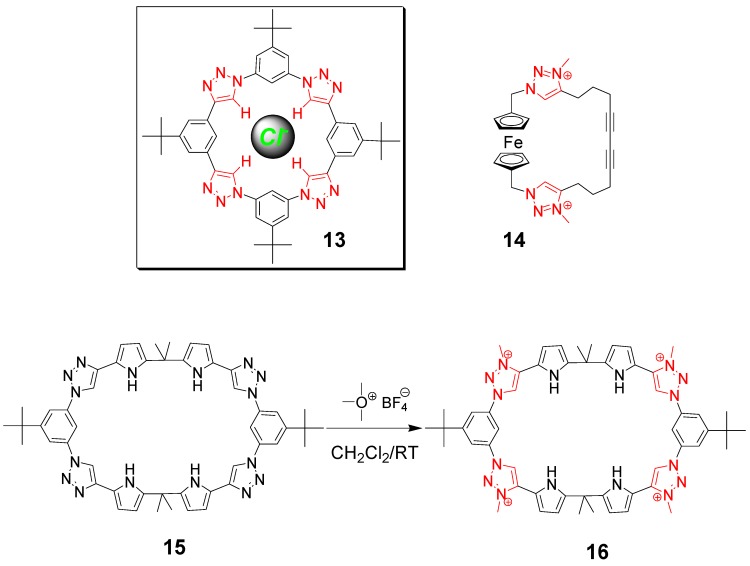
Triazole and triazolium-containing macrocycles for the binding of anions. In Red: triazole or triazolium amide surrogates as the key functionalities responsible for binding [59,61,62].

Recent reports have dealt with the study and synthesis of rigid macrocycles (often obtained by the use of aryl-containing spacing units) using CuAAC click chemistry, and the characterization of their host-guest binding properties [57,58]. It is, however, the work of Flood and coworkers that has demonstrated the possibility of using CuAAC click chemistry not only for the rapid, orthogonal and high yielding construction of shape-persistent macrocycles, but also for the utilization of the embedded triazoles resulting from the click process as amide surrogates for the binding of anions. The prototypical example is the highly preorganized, shape-persistent macrocycle **13** (Figure 3), in which the acidic C-H hydrogen of the four triazole units are perfectly positioned for the recognition of a spherical anion. Indeed, macrocycle **13** showed a very high binding affinity towards chloride anions in organic solvents [59]. Flood and coworkers have recently summarized the state of the art in this emerging subfield of research related to CuAAC click chemistry [60].

More recent reports have dealt with the possibility of increasing the hydrogen bonding capability of the neutral CH hydrogen bond donor motif (*i.e.*, triazole) by making it cationic (*i.e.*, triazolium moieties). The group of Beer has recently reported the synthesis and characterization of a clicked ferrocene-containing bis(triazole) macrocycle [61]. Alkylation gives the corresponding triazolium macrocycle (**14** in Figure 3), which strongly binds anions such as chloride and benzoate even in polar organic solvents. The authors presented good evidence (^1^H NMR titration experiment) that the high binding constant is favored by charge-assisted C–H·anion interactions, and they use the redox-active macrocycle **14** for electrochemical sensing of chloride ions in CH_3_CN solution.

The group of Sessler has reported the synthesis of rigid, shape persistent click macrocycle **15** designed to bind tetrahedral oxyanions (Figure 3). The macrocycle was obtained from triazole-containing precursors (obtained using CuAAC click chemistry), which were then cyclized by a condensation reaction with acetone to form the quaternary carbon atoms within the macrocyclic backbone. Postmodification of macrocycle **15** by reaction with the methylating agent trimethyloxonium tetrafluoroborate afforded the macrocycle **16**. The system incorporates neutral NH hydrogen bond donor groups (pyrrole), as well as cationic CH hydrogen bond donor motifs (triazolium moieties) [62]. The system displays a high selectivity for tetrahedral oxyanions, relative to monoanions and trigonal planar anions, in mixed polar organic-aqueous solvents. This selectivity is highly solvent dependent. Theoretical calculations were carried out in an effort to understand the influence of solvent on the intrinsic hydrogen bonding ability of the donor groups (pyrrole N–H and triazolium C–H). Macrocycle **16** can bind pyrophosphate and phosphate anions in the solid state as confirmed by X-ray diffraction analyses.

**Figure 4:**
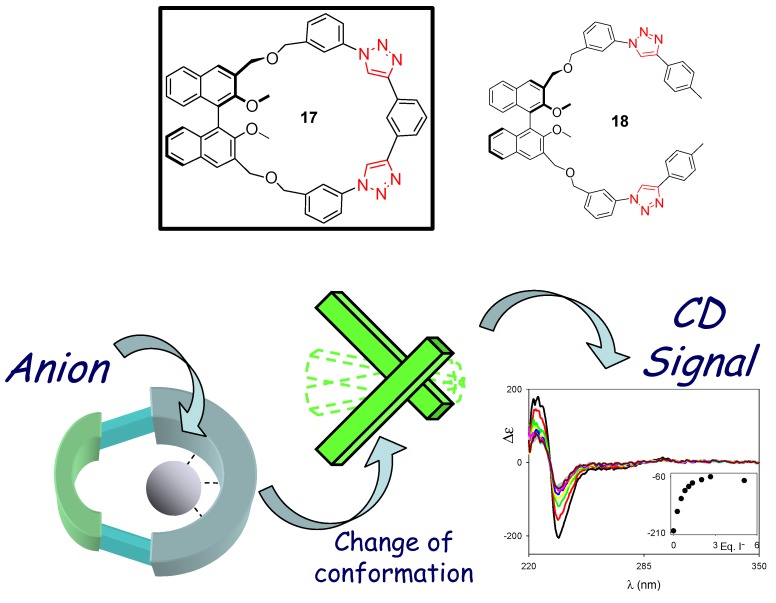
Structures of macrocyle **17** and control compound **18**, and (bottom) operating principles of the macrocyclic chiroptical sensor **17** in which the variation of the CD response of the Binol unit is the key sensing output [63].

We have recently reported the design and synthesis of a chiral click macrocycle behaving as a chiroptical sensor for the detection of halide anions (Figure 4) [63]. This work is part of our activity in the field of binaphthyl-based chiral macrocycles for chiroptical sensing and chiral nanostructuring [64,65,66,67,68,69,70,71,72,73,74,75,76,77,78,79,80]. The macrocycle **17** incorporates a binaphthyl (Binol) unit; the skeleton is not composed entirely of aromatic-type carbon atoms. In order to counterbalance the inherent distortion brought about by the binaphthyl units into the macrocyclic framework, sp^3^ carbon atoms, imparting a higher conformational freedom with respect to sp or sp^2^ hybridized carbon atoms, have been introduced.

Open control compound **18** was synthesized through classical CuAAC conditions. Macrocycle **17**, instead, needed to be synthesized by the use of nonclassical CuAAC conditions with the Cu(I) catalytic source directly introduced (and not generated *in situ*). As in previous examples [59], the CH hydrogen atoms of the triazole moiety are able to form hydrogen bonds with spherical anions, stabilizing the host-guest complex. The binding constants, measured in apolar organic solvents, are not remarkable, either for the macrocycle or the model compound. The system shows selectivity towards iodide, bromide and chloride over fluoride and carboxylate anions. The macrocycle (and not the control compound) is a “truly chiroptical” sensor for halide anions: strong signaling is generated when CD spectroscopy is used as the detection tool. The signalling mechanism is not a result of a specific recognition of the analyte with the CD reporter, the Binol moiety, but rather a secondary effect. The anions, held into the macrocyclic cavity, are in close proximity to the Binol moiety, and its conformational change generates the variation of the exciton-coupled signal classical signature of the Binol moiety.

## 4. Clicking Macrocycles to Form Mechanical Bonds

The CuAAC click reaction has been widely used for the preparation of interlocked molecules, such as catenanes and rotaxanes [81]. In the case of rotaxanes, the CuAAC reaction has been used mainly for the synthesis of the axle; subsequent threading, clipping or slipping procedures were utilized to produce the mechanically-interlocked product.

**Scheme 4:**
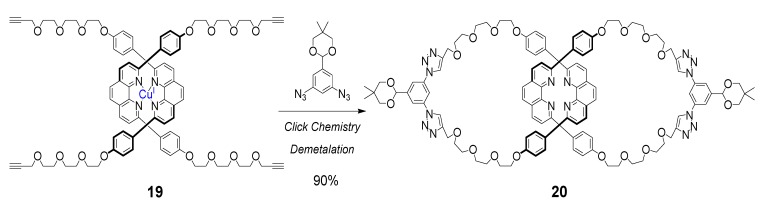
High yielding synthesis of “Sauvage-type” symmetrical catenane **20** [82].

In selected recent examples, the CuAAC reaction has been used for the ring-closing step of suitably positioned and entangled precursors. Megiatto and Schuster [82] have recently reported the synthesis of “Sauvage-type” catenanes, using click chemistry. “Sauvage-type” catenanes (Scheme 4) are synthesized by making use of a preorganized coordination complex in which two substituted phenanthroline units are optimally arranged around a Cu(I) template (structure **19** in Scheme 4). One of the challenges often encountered in the ring closure step of the pre-oriented phenanthroline moieties is that the complex is inherently unstable, and it can dissociate when subjected to temperature or solvent polarity changes, or to the presence of competing complexation ligands. The authors describe two different protocols based on Cu(I) template synthesis and “click” reactions for the synthesis of functionalized [2]catenanes. A straightforward procedure, involving high dilution conditions at high temperatures (70 °C), was developed for the synthesis of [2]catenanes bearing two identical peripheral groups in high yields (Scheme 4). The authors used “non classical” click chemistry conditions for the cyclization of precatenane **19**, by means of the direct introduction in the reaction mixture of a Cu(I) source and a base (DBU), but they also introduced sodium ascorbate. After demetalation, serendipitously occurring during the workup of the reaction, catenane **20** was obtained in an astonishing 90% isolated yield. For the preparation of non-symmetrically functionalized [2]catenanes, a milder protocol was developed, extending the methodology reported. The same authors reported the formation using click chemistry of a complex unsymmetrical material that would otherwise be impossible to synthesize, such as the porphyrin-C_60_ [2]catenane [83]. The introduction of peripheral functional groups into the catenane structure, using such high yielding methodology, paves the way to the use of [2]catenanes for the preparation of even more complex structures.

**Scheme 5:**
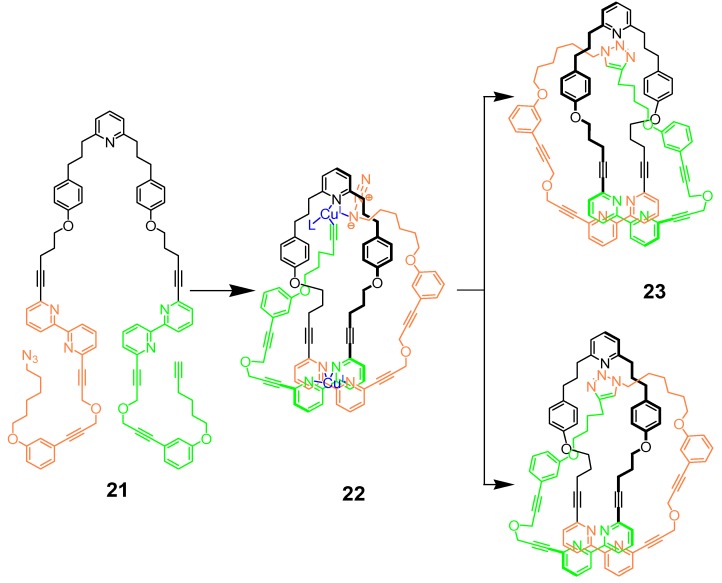
Synthesis of trefoil knot **23** by Leigh and co-workers [85].

In a recent article, Leigh and co-workers have applied CuAAC in a very elegant way for the synthesis of a trefoil knot [84,85]. The strategy has been dubbed as “the active-metal template”: in fact, Cu(I) ions both preorganize the knot precursor and act as the cyclization catalyst (Scheme 5). The open, flexible precursor **21** needs to be folded, before being cyclized into the trefoil knot **23**. The design of the molecule was chosen so that the arrangement of the ligands around the metal center forms the necessary entanglement to create the knot by coordination in a tetrahedral way to the bipyridine units (structure **22**). At the same time, a catalytic amount of the copper(I) ions complex the two unsymmetrical alkyne and azide extremities of the thread and the pyridine moiety, catalyzing the CuAAC click ring closing reaction between the two terminal moieties. After demetalation, a racemic mixture of left- and right-handed knots **23** was isolated in a yield of 24%, a very reasonable yield considering the synthetic challenge.

## 5. Cyclic Polymers Obtained by the CuAAC Click Reaction

Cyclic polymers represent a special class of macromolecules among the various possible polymeric architectures. Because of the absence of chain ends in cyclic polymers, different chain dynamics can strongly influence physical properties compared with linear analogues of the same mass [86]. Recently, therefore, large cyclic polymers have emerged from the realm of “scientific curiosities” to that of potentially useful macromolecular architectures for a variety of functional applications [87,88,89,90]. By virtue of the IUPAC definition [1], cyclic polymers can be considered as macrocycles. Polymers with (small or large) cyclic repeating units within the macromolecular backbone, cyclopolymers, have been known for some time [91,92,93,94,95].

**Scheme 6:**
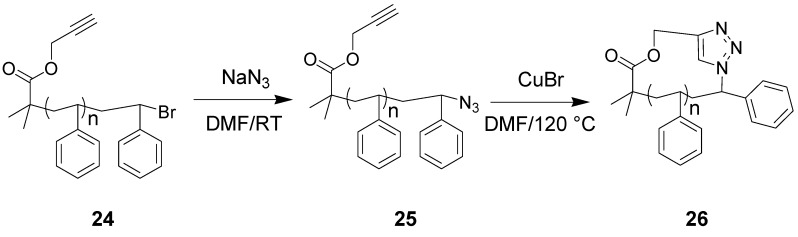
Terminal insertion of azide and “click” cyclization of polystyrene prepared via ATRP [96].

The group of Grayson has pioneered the application of CuAAC click chemistry to the realm of cyclic polymers. They prepared polystyrene macrocycles from ATRP (Atom Transfer Radical Polymerization) precursors containing a terminal alkyne (see Scheme 6) [96]. Polymer samples **24** were reliably obtained with low polydispersities (less than 1.2) and varying degrees of polymerization by virtue of the use of the controlled polymerization technique (ATRP). Further, the use of the ATRP methodology can afford polymers with tailored end-capping chain functionalities. The authors were able to transform the terminal bromide to an azide, to afford intermediate polymer **25**. Polymer **26** was then obtained by “click” cyclization with the pendant alkyne from the initiator; the concentration of the polymer samples has been carefully controlled to avoid polymer **25** to prefer condensation instead of cyclization. This route offers outstanding control over the size and polydispersity of the macrocyclic polymers, as well as being tolerant of a number of functional groups. More recently, the same group has reported the synthesis of well-defined toroidal macromolecules through a similar design [97].

The cyclization of linear polymers by coupling end-groups relies on the very slow feed of the uncyclized, linear polymer into a highly dilute reaction mixture of the CuAAC catalyst and solvents, leading to monocyclic polymer in very low concentrations over long periods of time (>10 h) and at high temperatures (>100 °C). The group of Montero has recently reported an alternative approach, in which the Jacobson-Stockmayer theory is utilized to predict the ratio of monocyclic polystyrene (c-PSTY) in a one-pot reaction at 25 °C [98]. Using the same substrates shown in Scheme 6, they found empirically, from experimental diffusion-controlled rate coefficients for cyclization and condensation of α,ω-polymer chains, that the Jacobson-Stockmayer theory is applicable for the CuAAC reaction. Therefore, the percentage of monocyclic polymer is independent of reaction rate parameters (such as catalysts concentration and temperature) and is only dependent on polymer concentration.

## 6. Summary and Perspectives

This review attempts to highlight recent achievements in the synthesis of cyclic structures by the use of the CuAAC click reaction. Perhaps the most useful demonstration of the utility and versatility of this reaction are given by the breadth of different chemical structures illustrated in this contribution. Cross-fertilization amongst different areas of chemistry has flourished owing to the utilization of the click reaction technique. In some of the selected cases, especially in the cases highlighted in Section 2 (untemplated cyclization of peptides in solution or in the solid phase), CuAAC represents the option of choice. We have also highlighted how sometimes CuAAC is now traditionally associated not only to triazoles, the organic functionality resulting from its application, but also from its transformation into more useful moieties for functional applications (triazolium salts). It is safe to anticipate a long life to this versatile reaction, in association with the synthesis of complex and functional molecular architectures. Given the low toxicity of triazoles [99], applications of CuAAC click chemistry in the field of functionalization of biopolymers towards innovative biomaterials and bio-based plastics can be envisaged.
